# Self-Reported And Objectively Recorded Colorectal Cancer Screening Participation In England

**DOI:** 10.1177/0969141315599015

**Published:** 2015-09-25

**Authors:** Siu Hing Lo, Jo Waller, Charlotte Vrinten, Jane Wardle, Christian von Wagner

**Affiliations:** Cancer Research UK Health Behaviour Research Centre, Department of Epidemiology and Public Health, University College London, Gower Street, London WC1E 6BT

**Keywords:** Colorectal cancer screening, self-reported uptake, objectively recorded uptake, over-reporting, under-reporting

## Abstract

**Objective:**

To compare self-reported with objectively recorded participation in Faecal Occult Blood testing (FOBt) colorectal cancer (CRC) screening in a national programme.

**Methods:**

Survey respondents living in England who were eligible for screening were asked in face-to-face interviews if they had ever been invited to do a CRC screening test, how many times they had been invited, and how many times they had participated. National Health Service (NHS) Bowel Cancer Screening Programme (BCSP) records were consulted for respondents who had consented to a record check. The outcome measures were ‘ever uptake’ (responded to ≥1 invitation), ‘repeat uptake’ (responded to ≥2 invitations), and ‘consistent uptake’ (responded to all invitations).

**Results:**

In the verified group, self-reported ever uptake was highly consistent with recorded ever uptake (87.0% vs. 87.8%). Among those who indicated that they had been invited more than once, self-reported repeat uptake was 89.8% compared with 84.8% recorded repeat uptake. Among those with more than one recorded invitation, self-reported *repeat* uptake was 72.7% compared with 77.2% recorded repeat uptake, and self-reported *consistent* uptake was 81.6% compared with 65.6% recorded consistent uptake.

**Conclusion:**

Our results suggest that people can accurately report whether they have ever taken part in CRC screening. The vast majority of those whose records were verified could also accurately report whether they had taken part in screening at least twice. They were somewhat less accurate in reporting whether they had responded to all screening invitations.

## Introduction

Self-reported measures are commonly used in health research. Objective behavioural measures are often difficult to obtain either because observation is not possible or due to limited accessibility of medical records for research purposes. Balancing research requirements with healthcare objectives can be challenging. There is evidence that using research questionnaires within the context of a national screening programme reduces Faecal Occult Blood test (FOBt) screening uptake.^1^ Concern exists about ‘over-reporting’ of health behaviours, particularly in studies of screening participation. Self-reported colorectal cancer (CRC) screening uptake tends to be higher than objectively recorded uptake for all CRC screening modalities, and especially for FOBt screening.^2,3^ Although over-reporting of CRC screening is well-documented, the phenomenon is not well-understood. A recent study showed that social desirability, a common explanation for false survey responses, was not associated with accuracy of self-reported CRC screening.^4^

The existing literature has limited applicability to organized, national screening programmes; 87% of available studies comparing self-reported with objectively recorded CRC screening uptake were conducted in United States, where screening is often self-initiated, recommendations include several different tests,^2,5^ and there are no standardized audit processes for screening records.^2^ Variation in the quality of medical records may contribute to discrepancies between self-reported and recorded screening participation. Elsewhere, population-based programmes are recommended by institutions (eg. the European Union) and are therefore becoming increasingly common.^6^ Previous findings, therefore, may not generalize to countries with national screening programmes, a single screening modality, and centralized medical record databases. Previous studies have also focused on whether people can recall ‘ever’ participating in screening, or accurately report the timing of their most recent screen (ie. whether they are ‘up to date’).^2,3,7^ These questions, while important, do not capture all relevant aspects of adherence. Repeated, consistent participation is important for FOBt screening, due to low test sensitivity.^8,9^ One complicating factor in self-reports of repeat uptake and repeated consistent uptake is recall of multiple screening episodes. Inaccurate self-report could be the result of failing to correctly report the number of episodes or participation in these episodes. Accuracy of self-reported repeat uptake and repeated consistent uptake should therefore also be assessed.

We compared self-reported with recorded CRC screening participation among survey respondents in England, who had consented to having their screening records retrieved. We aimed to compare self-reported with recorded ever, repeat, and consistent uptake in an organized, national screening programme.

## Methods

CRC screening in England is organized by the National Health Service (NHS) Bowel Cancer Screening Programme (BCSP). All men and women aged 60-70 (recently extended to 74) are mailed a free guaiac FOBt kit, with a freepost return envelope, every two years. Around 4-6%^10^ are requested to complete more than one test within any one screening round, mostly due to a weak positive test result.

**Figure 1.**
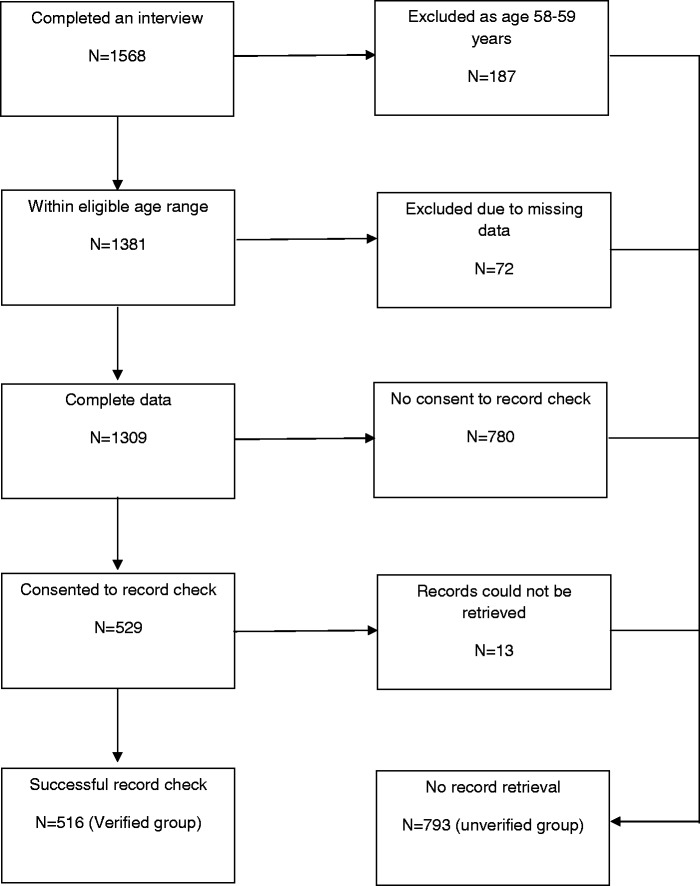
Sample sizes, inclusion and exclusion criteria.

A population-based survey conducted in Great Britain between January and March 2014 (TNS Research International) used 2011 Census small-area statistics and the Postcode Address File (stratified by social grade and Government Office Region) for random location sampling selection, setting quotas at each location for age, gender, children in the home, and working status. Interviewers visited each address and invited householders to participate in face-to-face interviews using computer-assisted personal interviewing. Respondents aged 58-70, resident in England, with no history of CRC, were included in the cancer screening survey (n = 1568) (see Figure 1). Respondents aged 58-59 (n = 187) were excluded from our study because they were not eligible for NHS BCSP FOBt screening at the time of the interview. Cases with missing values for self-reported screening uptake (ie. ‘refused’ or ‘don’t know’; n = 72) were also excluded, producing a final sample of 1,309. At the end of the survey, respondents were invited to consent to their screening records being accessed to verify their past and future screening participation (see Appendix). They could give the consent form to the interviewer, or return it by post. Of 1,309 survey respondents, 529 consented to the record check, and for 516 of these (the ‘verified group’, 39.4% of the total sample) screening records were retrieved. Non-retrieval of records was mostly due to illegible handwriting or incorrect birth dates (a few respondents gave the date of the survey). The verified group was compared with the 793 respondents for whom records were not retrieved (the ‘unverified group’, 60.6% of the sample). Ethical approval for this study was obtained from the NHS (REC 13/NW/0707).

A dichotomous variable was created to indicate whether respondents were in the unverified group [0] or the verified group [1].

Respondents were asked if they had ever been invited to do the stool test for the NHS Bowel Cancer Screening Programme. They were told that it was also known as the Faecal Occult Blood Test and that it would have arrived by post. If their answer was affirmative, further questions were asked to determine the number of times they had been invited and the number of times they had participated. An overview of uptake definitions is provided in Table 1. ‘Self-reported ever uptake’ was a dichotomous variable with the categories ‘never uptake’ (never completed a screening test kit *OR* never invited) and ‘ever uptake’ (completed ≥ 1 test kit).

**Table 1.** Definitions of uptake measures.

	Inclusion criteria	Self-reported	Recorded
Never screened	All respondents aged 60–70	Never invited OR never completed	Invited at least once AND never screened
Ever uptake	All respondents aged 60–70	Completed ≥1 test kit	Screened for ≥1 invitations
Repeat uptake I	Respondents aged 60–70 who self-reported ≥2 invitations	Completed ≥2 test kits	Screened for ≥2 invitations
Repeat uptake II	Respondents aged 60–70 with ≥ 2 recorded invitations	Complete ≥2 test kits	Screened fo ≥2 invitations
Consistent uptake	Respondents aged 60–70 with ≥2 recorded invitations	No. of completed test kits = No. of test kits received	No. of episodes screened = No. of screening invitations

Two possible denominators for self-reported repeat uptake were used. Respondents who *self-reported* having been invited to participate more than once were divided into two categories: ‘no repeat uptake’ (completed no or one test kit) and ‘repeat uptake’ (completed ≥ 2 test kits) - we call this measure ‘self-reported repeat uptake I’. This first denominator is the one that would be used in studies using survey data only, and allowed us to explore the accuracy of these survey responses. Among respondents in the verified group who had more than one *recorded* invitation, we examined ‘self-reported repeat uptake II’ (using the categories described above) and ‘self-reported consistent uptake’. Categories for self-reported consistent uptake were: ‘never or inconsistent uptake’ (number of completed test kits < number of test kits received) and ‘consistent uptake’ (number of completed test kits = number of test kits received). This second denominator was only available for respondents in the verified group, but did not rely on respondents accurately recalling the number of screening invitations they had received. It allowed us to explore the extent of under- and over-reporting of repeat participation among those who had been invited twice, irrespective of whether they could recall the invitations.

Similar to self-reported uptake, a ‘recorded ever uptake’ variable, a ‘recorded repeat uptake’ variable with recorded repeat invitation as a denominator, and ‘recorded consistent uptake’ were generated for respondents in the verified group. For screening verification, ID numbers, names, postcodes and dates of birth were sent in July 2014 to the NHS BCSP, who returned ID numbers, the number of times each individual had been invited for screening, and whether they had responded to each screening invitation.

Sociodemographic variables age, sex, marital status (married vs. widowed, divorced or separated vs. single), ethnicity (white/ non-white), and social grade were recorded. Social grade was measured using the National Readership Survey social grade classification system, which is based on occupation (or previous occupation if retired): A (higher managerial, administrative or professional), B (intermediate managerial, administrative or professional), C1 (supervisory, clerical or junior managerial, administrative or professional), C2 (skilled manual), D (semi-skilled or unskilled manual) or E (state pensioners, casual/ lowest grade workers or unemployed with state benefits only). The occupational status of the chief wage earner in the household was used if a respondent did not work and was not retired.

Sample characteristics of the verified and unverified groups were compared. Logistic regression analysis was used to examine bivariate and multivariable associations between record verification (outcome variable) and sociodemographics and self-reported uptake (explanatory variables). Self-reported uptake rates were described for the total sample, the unverified group, and the verified group. Recorded uptake rates for the verified group were also presented. For the verified group, self-reported uptake and recorded uptake were compared. Concordance between self-reported and recorded uptake, kappa statistics, and McNemar’s chi-square statistics are reported alongside the proportion of ‘accurate reporters’ (self-report equals recorded uptake), ‘under-reporters’ (self-reported uptake is lower than recorded uptake) and ‘over-reporters’ (self-reported uptake is higher than recorded uptake). All analyses used pairwise deletion and were conducted using Stata Version 13SE.^11^

## Results

Table 2 shows the sample characteristics for the included respondents. The distribution of social grade was slightly skewed, with an overrepresentation of more deprived groups (5.1% in highest grade v 22.5% in lowest). In line with the lower prevalence of ethnic minorities among older age groups in the national population of England,^12^ only 4.1% of respondents were non-white. Of the total sample, 39% were in the verified group. Table 3 shows that the odds of being in the verified group decreased with lower social grade (p < .001), and being non-white was also associated with lower odds of being in the verified group than being white (p < .01). There were no statistically significant differences in age, gender or marital status in record verification. Those who self-reported ‘ever’ having taken part were also more likely to be in the verified group (p < .001; Table 3). This translated to 87.0% self-reported ever uptake in the verified group compared with 57.9% for those in the unverified group (Table 4). Multivariable analysis showed that being in the verified group was independently associated with higher social grade, white ethnicity, and self-reported ‘ever’ participation in FOBt screening (Table 2).

**Table 2.** Total sample characteristics (n=1309).

	Total sample
	% (n)
Total	100% (1309)
Self-reported ever uptake	
Never	30.6% (401)
Ever	69.4% (908)
Age	
60-64	42.1% (551)
65-69	57.9% (758)
Gender	
Men	50.7% (664)
Women	49.3% (645)
Marital status	
Married	65.0% (851)
Div./ sep./ widowed	26.2% (343)
Single	8.8% (115)
Ethnicity	
White	96.0% (1256)
Non-white	4.1% (53)
Social grade	
A (highest grade)	5.0% (66)
B	20.4% (267)
C1	22.1% (289)
C2	18.3% (240)
D	11.6% (152)
E (lowest grade)	22.5% (295)

a As a continuous variable; *p < .05, **p < .01, ***p < .001.

**Table 3.** Percentage (n) of respondents from each demographic group who were in the unverified and the verified groups, and logistic regression analysis of predictors of record verification.

	Unverified group	Verified group	Bivariate results		Multivariable results	
	% (n)	% (n)	Unadjusted OR	95% CI	Adjusted OR	95% CI
Total	60.6% (793)	39.4% (516)				
60-64	60.8% (335)	39.2% (216)	1.00 (ref.)		−	
65-69	60.4% (458)	39.6% (300)	1.02	0.81 – 1.27		
Gender						
Men	59.3% (394)	40.7% (270)	1.00 (ref.)		−	
Women	61.9% (399)	38.1% (246)	0.90	0.72 – 1.12		
Marital status						
Married	59.7% (508)	40.3% (343)	1.00 (ref.)		−	
Div./ sep./ widowed	62.1% (213)	37.9% (130)	0.90	0.70 – 1.17		
Single	62.6% (72)	37.4% (43)	0.88	0.59 – 1.32		
Ethnicity						
White	59.6% (748)	40.5% (508)	1.00 (ref.)		1.00 (ref.)	
Non-white	84.9% (45)	15.1% (8)	0.26**	0.12 – 0.56	0.42*	0.19 – 0.93
Social grade (1-6)						
A – 1 (highest grade)	47.0% (31)	53.0% (35)	0.78^a^***	0.73 – 0.84	0.81^a^***	0.75 – 0.88
B – 2	49.1% (131)	50.9% (136)				
C1 – 3	57.8% (167)	42.2% (122)				
C2 – 4	60.0% (144)	40.0% (96)				
D – 5	67.8% (103)	32.2% (49)				
E – 6 (lowest grade)	73.6% (217)	26.4% (78)				
Self-reported ever uptake						
Never	83.3% (334)	16.7% (67)	1.00 (ref.)		1.00 (ref.)	
Ever	50.6% (459)	49.5% (449)	4.88***	3.64 – 6.54	4.47***	3.32 – 6.01

a As a continuous variable; *p < .05, **p < .01, ***p < .001.

**Table 4.** Percentage (n) self-reported vs. recorded uptake in the verified group and the unverified group.

	Total sample		Unverified group		Verified group			
	N	Self-reported	N	Self-reported	N	Self-reported	N	Recorded
**Ever uptake**							
	1309	69.4% (908)	793	57.9% (459)	516	87.0% (449)	516	87.8% (453)
**Repeat uptake I**							
*self-reported ≥ 2 invitations*	733	82.5% (605)	389	76.1% (296)	344	89.8% (309)	341^a^	84.8% (289)
**Repeat uptake II**							
*recorded ≥ 2 invitations*	−	−	−	−	451	72.7% (328)	451	77.2% (348)
**Consistent uptake**							
*recorded ≥ 2 invitations*	−	−	−	−	451	81.6% (368)	451	65.6% (296)

a 3 respondents reported having received two or more test kits, but had only one recorded screening round.

**Figure 2.**
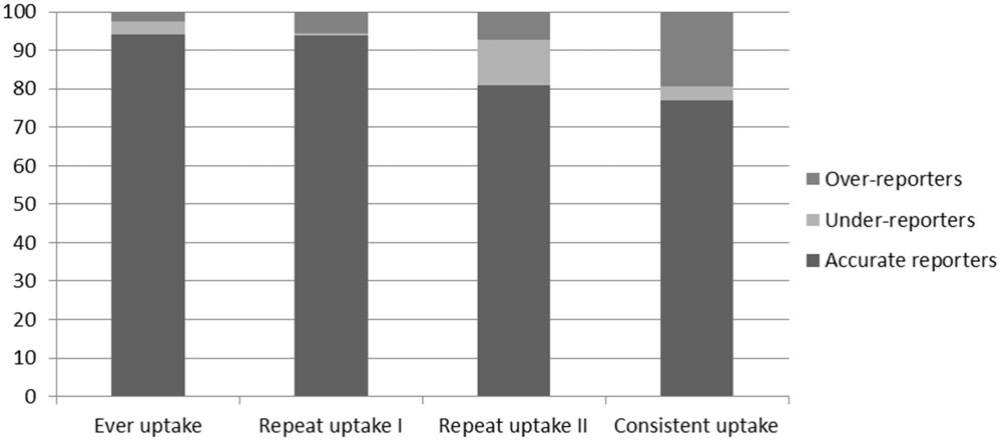
Percentage accurate reporters, under-reporters and over-reporters for ever, repeat and consistent uptake in the verified group.

Self-reported ever uptake was 87.0% compared with a recorded uptake of 87.8% (Table 3), corresponding to 94.2% agreement and a kappa of 0.74 (Table 4). Agreement between self-reported and recorded uptake was therefore ‘substantial’ for ever uptake.^13^ Slightly higher recorded uptake would be expected even if self-reported uptake was completely accurate, due to the timing of the record check (See Discussion). As also illustrated in Figure 2, Table 5 shows that the percentage of under-reporters (3.3%) and over-reporters (2.5%) for ever uptake were relatively small, and similar in size (χ^2 ^= 0.53, non significant).

**Table 5.** Accuracy of self-reported ever, repeat and consistent uptake in the verified group (total n = 516).

	Sample (n)	Accurate % (n)	Under-reporters % (n)	Over-reporters % (n)	Kappa	McNemar’s Test	
						χ^2^	p-value
**Ever uptake**	516	94.19% (486)	3.29% (17)	2.52% (13)	0.74	0.53	p = 0.47
in the total verified group							
**Repeat uptake I**	341	93.84% (320)	0.59% (2)	5.57% (19)	0.72	13.76	p < .001
in those self-reporting ≥ 2 invitations						
**Repeat uptake II**	451	80.93% (365)	11.75% (53)	7.32% (33)	0.49	4.65	p = 0.03
in those with ≥ 2 recorded invitations						
**Consistent uptake**	451	76.94% (347)	3.55% (16)	19.51% (88)	0.43	49.85	p < .001
in those with ≥ 2 recorded invitations						

* p < .05, **p < .01, ***p < .001.

Among those who *self-reported* having received at least two screening test kits (repeat uptake I), self-reported repeat uptake was 89.8% compared with a recorded repeat uptake of 84.8% (Table 4). This corresponded to 93.8% agreement and a kappa of 0.72 (Table 5), indicating a ‘moderate’ to ‘substantial’ level of agreement.^13^ However, the percentage of over-reporters (5.6%) was larger than that for under-reporters (0.6%), suggesting a degree of over-reporting of repeat uptake among those who self-reported having received at least two kits (χ^2 ^= 13.76, p < .001; Table 5 - Figure 2).

Among those who had at least two *recorded* screening invitations (repeat uptake II), self-reported repeat uptake (72.7%) was lower than recorded uptake (77.2%; Table 4), corresponding to 80.9% agreement and a kappa of 0.49 (Table 5), suggesting a ‘fair’ to ‘moderate’ level of agreement.^13^ In contrast to repeat uptake based on self-reported repeat invitation, the proportion of under-reporters (11.8%) was larger than the proportion of over-reporters for those who had at least two recorded invitations (7.3%; χ^2 ^= 4.65, p < .05; Table 5 - Figure 2). Among under-reporters, 51 out of 53 (96%) had reported receiving and completing only one test kit (*not shown in table*). This suggests that under-reporting was a result of underestimating the number of screening invitations received, not incorrect reporting of non-response to an invitation received.

Within the group of respondents who had at least two recorded invitations, self-reported consistent uptake (defined as having responded to all invitations irrespective of the number of invitations) was 81.6% compared with 65.6% recorded consistent uptake (Table 4). This corresponded to 76.9% agreement and a kappa of 0.43, indicating a ‘fair’ to ‘moderate’ level of agreement.^13^ The proportion of over-reporters (19.5%) was larger than the proportion of under-reporters (3.6%), indicating a significant degree of over-reporting (χ^2 ^= 49.85, p < .001; Table 5 – Figure 2).

## Discussion

This study compared self-reported and objectively recorded CRC screening uptake in the context of the national screening programme in England. Self-reported ever uptake corresponded very well with recorded ever uptake among survey respondents for whom screening records were verified, suggesting that survey respondents are able to reliably report ever uptake, but under-reporting of the number of received invitations and over-reporting of consistent participation was also demonstrated. Under-reporting of the number of received invitations (and by implication, under-reporting of repeat uptake) was mainly due to respondents reporting having been invited and taken part once, despite having been invited and taken part at least twice. Self-reported repeat uptake was much more accurate among verified respondents who accurately reported having been invited at least twice, and over reporting was small in this group. Over-reporting of consistent participation was more substantial, suggesting that the likelihood of failing to report one or more missed screening episode(s) increases with the number of invitations.

A majority were able to report accurately whether they had participated in screening at least twice, and whether they had responded to every screening invitation received. This suggests that self-reported measures of ever uptake (“have you ever done the stool test?”), repeat uptake (“have you done the stool test at least twice?”), and consistent uptake (“have you done the stool test every time?”) can be useful when assessing CRC screening uptake. Reliable self-report measures are important because medical records are difficult to access outside healthcare settings, as demonstrated by the fact that only 39% of participants in this study consented to the record check and had their records subsequently verified.

Self-reported uptake and recorded uptake could only be compared for survey respondents in the verified group, who had a higher occupational social grade, were more likely to be white, and were more likely to report ever having taken part in screening than those in the unverified group. Because lack of consent was the main reason for records not being available, the sample for whom we could compare self-reported and recorded uptake was not representative of the population. The requirement for written consent may have deterred respondents with low literacy, or whose first language was not English. This may explain why higher social grade and white ethnicity were associated with increased odds of being in the verified group. Previous research^4^ and the lower self-reported uptake rates among unverified respondents suggest that social desirability is unlikely to have been the reason for refusing consent. Another limitation of the present study was the small number of ethnic minority individuals in the verified group. Although consistent with the low prevalence of ethnic minorities in older age groups in England,^14^ this prevented further examination of possible ethnic differences in reporting of uptake.

The study results should be interpreted in the light of a small, but expected mismatch between self-reported and recorded uptake, even if self-report were perfectly accurate. The NHS BCSP system does not record uptake status until 12 weeks after an invitation is sent, but the time elapsed between the survey data collection and the record check was three to six months. A small number of respondents could therefore have completed the screening test after they took part in the survey, implying that recorded uptake should be slightly higher than self-reported uptake if self-reported uptake is accurate. Furthermore, the survey measures used in the present study asked about the number of screening *kits* received and completed, while the NHS BCSP data record the number of screening invitation *rounds* an individual has completed adequately. As a small minority (4-6%) of screening participants are requested to complete more than one test kit within a single invitation round,^10^ any comparison of self-reported uptake and recorded uptake that relies on the number of test kits being equal to the number of screening rounds should show a small discrepancy, even if self-report is accurate. An individual may accurately report having completed two kits, while the screening records will only show the adequate completion of that screening round. This does not mean that self-report measures of FOBt screening cannot be clinically meaningful. To reap the full benefits of FOBt screening, participants should screen repeatedly, ideally every time they are invited for screening. In the present study, the majority of verified respondents could accurately report whether they had taken part more than once and (with slightly less accuracy) whether they had ever failed to respond to a screening invitation. This should be sufficient to relate self-report measures to meaningful clinical outcomes, without having to rely on medical records for the precise number of test kits or screening rounds completed.

Our research suggests that ‘ever’ screening participation can be a very reliable behavioural outcome for studies that use self-reported screening uptake, and that most survey respondents can accurately report whether they have taken part more than once, and whether they have taken part consistently. These study findings may have implications for measurement of breast and cervical screening participation, which also require repeated screening with long intervals between screening episodes.

## Supplementary Material

Supplementary material

## References

[1] Watson J Shaw K MacGregor M Use of research questionnaires in the NHS Bowel Cancer Screening Programme in England: impact on screening uptake. Journal of Medical Screening. 2013; 20: 192–7. 24177175 10.1177/0969141313511447

[2] Dodou D, de Winter JC. Agreement between self-reported and registered colorectal cancer screening: a meta-analysis. *European Journal of Cancer Care*. 2015;**24**:286–98. 10.1111/ecc.12204 24754544

[3] Rauscher GH Johnson TP Cho YI Walk JA Accuracy of self-reported cancer-screening histories: A meta-analysis. Cancer Epidemiology Biomarkers & Prevention. 2008; 17: 748–57. 10.1158/1055-9965.EPI-07-2629 18381468

[4] Vernon SW Abotchie PN McQueen A Is the Accuracy of Self-Reported Colorectal Cancer Screening Associated with Social Desirability? Cancer Epidemiology Biomarkers & Prevention. 2012; 21: 61–5. 10.1158/1055-9965.EPI-11-0552 PMC3268255 22144501

[5] Beebe TJ Ziegenfuss JY Jenkins SM Lackore KA Johnson TP Survey mode and asking about future intentions did not impact self-reported colorectal cancer screening accuracy. BMC Medical Research Methodology. 2014; 14: 19–19. 24499399 10.1186/1471-2288-14-19 PMC3918109

[6] Wittmann T Stockbrugger R Herszenyi L New European Initiatives in Colorectal Cancer Screening: Budapest Declaration. Digestive Diseases. 2012; 30: 320–2. 22722559 10.1159/000337006

[7] Howard M Agarwal G Lytwyn A Accuracy of self-reports of Pap and mammography screening compared to medical record: a meta-analysis. Cancer Causes & Control. 2009; 20: 1–13. 18802779 10.1007/s10552-008-9228-4

[8] Soares-Weiser K, Burch J, Duffy S, et al. Diagnostic Accuracy And Cost-Effectiveness Of Faecal Occult Blood Tests (FOBT) Used In Screening For Colorectal Cancer: a Systematic Review. York, United Kingdom: University of York, 2007. 10.1258/096914107782066220 17925085

[9] Steele RJC McClements PL Libby G Carey FA Fraser CG Patterns of uptake in a biennial Faecal Occult Blood Test (FOBT) screening programme for colorectal cancer. Colorectal Disease. 2014; 16: 28–32. 24034143 10.1111/codi.12393

[10] Lo SH Halloran S Snowball J Colorectal cancer screening uptake over three biennial invitation rounds in the English bowel cancer screening programme. Gut. 2015; 64: 282–91. 24812001 10.1136/gutjnl-2013-306144 PMC4316922

[11] StataCorp. Stata Statistical Software: Release 13. College Station, TX: StataCorp LP, 2013.

[12] Office of National Statistics. Detailed Characteristics for England and Wales, March 2011, 2013.

[13] Viera AJ Garrett JM Understanding interobserver agreement: the kappa statistic. Family Medicine. 2005; 37: 360–3. 15883903

[14] Office of National Statistics. Focus on Ethnicity and Identity, Summary Report: Office for National Statistics2005 25th March 2005.

