# Supplementary material for: Self-Reported And Objectively Recorded Colorectal Cancer Screening Participation In England
Source: J Med Screen. 2015 Sep 25;23(1):17–23. doi: 10.1177/0969141315599015 (PMC4741296; doi:10.1177/0969141315599015)
Supplement: Supplementary material [file MSC599015_supplementary_file.pdf]

PARTICIPANT INFORMATION SHEET, VERSION 1.3, 2nd JANUARY, 2014

Your participation in the NHS Bowel Cancer Screening Programme

Today's survey and our research

Thank you very much for answering our questions about bowel cancer screening today.

We are researchers from University College London (UCL) funded by Cancer Research UK to find out more about participation in the NHS Bowel Cancer Screening Programme.

The main questions we would like to answer with the responses from you today are:

1) What do people think about bowel cancer screening?
2) Who takes part in bowel cancer screening?

To get more information on the second question we would like to **check your participation in the NHS Bowel Cancer Screening Programme over time.** TNS will not be involved in this part of the research and will not store any NHS data.

Please note that we will NOT be asking for information about the results of any screening tests or your participation in any other cancer screening programmes. Your responses will be kept strictly anonymous.

All research involving the NHS is looked at by an independent Research Ethics Committee to protect your interests. This study has been reviewed and given a favourable opinion by an NHS Research Ethics Committee.

How can we check your response to bowel cancer screening invitations?

To check your response to screening invitations, we will need to send your name, date of birth and postcode together with an ID number to the NHS Bowel Cancer Screening Programme, e.g.:

ID number	Last name	First name	DOB	Postcode
1	Jones	Mary	01/ 12 / 1950	WC1 7HB

The NHS Bowel Cancer Screening Programme will then send us information about whether and when you have been invited and if so, whether and when you have accepted screening invitations with your ID number, but WITHOUT your name, date of birth or postcode, e.g.:

ID no.	1st invitation date	1st response	1st response date	2nd invitation date	2nd response	2nd response date
1	01/01/2011	Yes	20/01/2011	20/01/2013	No	-

What happens if I AGREE to UCL checking my screening participation?

If you agree to us checking your response to screening invitations, **we will not contact you again**, unless you have explicitly indicated that you are happy to be re-contacted for future studies. We will check your response to any bowel cancer screening invitations once in 2014 and another time in 2016. The aim is to publish the anonymised study results in medical journals over the next 2 to 5 years. The interviewer will leave you a copy of this information sheet in case you have questions later. If you change your mind, you may contact the study researcher to withdraw your consent (see contact details below).

The researchers are bound by professional data security and ethics guidelines. This means that they need to treat the data confidentially. They also need to restrict access to the survey responses and information about your response to screening invitations by using password-protected files. Only researchers involved in this study would have access to the relevant passwords. In addition, all person-identifiable details have to be kept in a separate file from the survey responses and information about your response to screening invitations. The file with person-identifiable details will be destroyed by 2016 once we have collected all relevant information about your participation in the NHS Bowel Cancer Screening Programme.

What happens if I DON'T AGREE to UCL checking my screening participation?

It is up to you to decide whether or not you are happy for us to ask for information about your response to screening invitations from the NHS Bowel Cancer Screening Programme. Your decision will NOT affect the standard of care you receive now or in the future.

The interviewer will leave you a copy of this information sheet in case you change your mind or have questions later.

I need to think about this; can I make up my mind later?

Yes, the interviewer will leave you a copy of this information sheet. You can respond to our request using the reply form and freepost envelope at your earliest convenience.

Contact details

You can contact the study researcher directly with any questions, comments or complaints:

Dr Siu Hing Lo
Department of Epidemiology and Public Health, University College London
1-19 Torrington Place, London WC1E 6BT
Tel: 020 3108 3099 E-mail: s.lo@ucl.ac.uk

The normal NHS complaints mechanisms are also available to you.
Details can be obtained from the Department of Health website: http://www.dh.gov.uk

REPLY FORM

I give permission for UCL researchers to contact the NHS Bowel Screening Programme to check my response to bowel cancer screening invitations.

[ ] YES *Please complete details in box below*

[ ] NO *Thank you for reading the information (no further details are required)*

IF YES

Please tick (✔) boxes below

I confirm that I have read and understood the information leaflet dated 14th October, 2013, version 1.2 for the above study and have had the opportunity to ask questions. ☐

I confirm that I have had sufficient time to consider whether or not to be included in the study. ☐

I understand that my personal details (i.e. name, date of birth, postcode) and relevant data about my participation in the NHS Bowel Cancer Screening Programme will only be accessed by responsible individuals from University College London and the NHS Bowel Cancer Screening Programme where it is relevant to my taking part in this research. ☐

Please use BLOCK CAPITALS

Family name (surname):

First name:

Date of birth (date/month/year):

UK postcode:

Signature

____

THANK YOU VERY MUCH FOR YOUR HELP WITH OUR RESEARCH
